# Pumpkin Seeds for Tape-Worm

**Published:** 1855-01

**Authors:** 


*Pumpkin Seeds for Tape Worm.*

A sufficient number of cases have been reported to show that the emulsion made of Pumpkin seeds possesses some real efficacy as a remedy for the expulsion of *tape-worm.* The usual mode of using it, is to remove the rind from the seeds, bruise the latter in a mortar, and infuse them in water, in the proportion of two ounces of the seeds to half a pint of water. After standing a few hours the whole assumes the form of an emulsion or thick mucilage. The whole of this should be taken by an adult, in the morning fasting; and in two or three hours after, sufficient castor oil should be given to move the bowels freely. If this does not cause expulsion of the worm, repeat the same the following morning.

